# Combination of Polymer Gate Dielectric and Two-Dimensional Semiconductor for Emerging Field-Effect Transistors

**DOI:** 10.3390/polym15061395

**Published:** 2023-03-10

**Authors:** Junhwan Choi, Hocheon Yoo

**Affiliations:** 1 Department of Chemical Engineering, Dankook University, 152 Jukjeon-ro, Yongin 16890, Republic of Korea; 2 Department of Electronic Engineering, Gachon University, 1342 Seongnam-daero, Seongnam 13120, Republic of Korea

**Keywords:** polymer dielectric, polymer thin film, atomically thin material, 2D semiconductor, gate dielectric, field-effect transistor

## Abstract

Two-dimensional (2D) materials are considered attractive semiconducting layers for emerging field-effect transistors owing to their unique electronic and optoelectronic properties. Polymers have been utilized in combination with 2D semiconductors as gate dielectric layers in field-effect transistors (FETs). Despite their distinctive advantages, the applicability of polymer gate dielectric materials for 2D semiconductor FETs has rarely been discussed in a comprehensive manner. Therefore, this paper reviews recent progress relating to 2D semiconductor FETs based on a wide range of polymeric gate dielectric materials, including (1) solution-based polymer dielectrics, (2) vacuum-deposited polymer dielectrics, (3) ferroelectric polymers, and (4) ion gels. Exploiting appropriate materials and corresponding processes, polymer gate dielectrics have enhanced the performance of 2D semiconductor FETs and enabled the development of versatile device structures in energy-efficient ways. Furthermore, FET-based functional electronic devices, such as flash memory devices, photodetectors, ferroelectric memory devices, and flexible electronics, are highlighted in this review. This paper also outlines challenges and opportunities in order to help develop high-performance FETs based on 2D semiconductors and polymer gate dielectrics and realize their practical applications.

## 1. Introduction

Two-dimensional (2D) semiconducting materials are atomically layered structures, a few nanometers or less in thickness [1,2]. Their atomically thin nature allows electrons to be transported in the 2D in-plane direction. Graphene was one of the first studied 2D materials. Although it is unsuitable for transistor applications because it has no bandgap [3,4,5], its properties, such as excellent electron and hole mobility and carrier density modulation through gating, give it unique band structures—i.e., Dirac cones—which are particularly interesting for optical applications [6,7]. As post-graphene 2D semiconductor materials, transition metal dichalcogenides (TMDs) are considered promising alternatives [8,9,10,11,12]. TMDs are based on an MX_2_ sandwiched structure, where M is a transition metal atom, such as Mo, W, or Pt, and X is a chalcogen atom, such as S, Se, or Te. These TMDs are atomically thin structures. For example, molybdenum disulfide (MoS_2_) [13,14,15], the most frequently investigated TMD material, has a thickness of only 6.5 Å as a monolayer. In addition to this atomically thin structural benefit, TMDs can potentially offer high carrier mobility [16,17], sharp subthreshold switching swings [18,19], and mechanical stress endurance [20,21,22], so they have rapidly emerged as the core of next-generation electronic devices.

Improving transistor characteristics requires consideration of semiconductors and the dielectric part because the capacitance of the gate dielectric determines the operating voltage [23,24]. Moreover, the dielectric surface characteristics determine the threshold voltage (*V*_T_) [25,26] and charge trapping behaviors [27,28] because the interface between the gate dielectric and the semiconductor layer is the charge transport path. Compared to inorganic dielectric materials, which are typically employed for 2D semiconductor FETs [29,30], polymer dielectrics have distinct advantages when they are combined with 2D semiconducting materials. With functional molecular structures and inherent flexibility [31,32,33,34], polymer-based gate dielectrics offer high compatibility with thin-film semiconductors and polymer substrates. Furthermore, compared to inorganic dielectrics, which require a higher thermal budget, polymers can be processed using more energy-efficient methods, such as solution processing [27,35,36] or low-temperature vapor-phase synthesis techniques [37,38]. These relatively simple processing methods not only make polymer dielectric films cost-effective but also enable the formation of ultrathin polymer films on top of 2D materials, which is difficult to achieve with inorganic dielectrics [39,40]. In fact, since the chemisorption is restricted to the top of the dangling bond-free surface of 2D materials in atomic layer deposition (ALD), inorganic dielectric layers typically require ultrathin seed layers, which makes the fabrication process complicated [29,41]. More importantly, compared to most inorganic dielectrics based on oxides, the molecular functionality of a polymer can induce interface dipoles and charge-transfer doping, which provides control over electrical properties [42,43,44].

With these clear advantages, polymer dielectric materials have been extensively investigated in order to develop 2D semiconductor FETs, as we revisit in this study. In an FET operation, the drain current (*I*_D_) at a given gate voltage (*V*_G_) is proportional to the capacitance of the dielectric layer, which is proportional to the dielectric constant (*k*) and inversely proportional to the thickness [45]. Thus, improving the dielectric constant, as well as reducing the thickness of the polymer dielectric film, is important to enhance output current at low operating voltages. On the other hand, the off current (*I*_off_) should be kept low to minimize standby power consumption and to maximize the current on/off ratio (*I*_on_/*I*_off_) [46]. Since the dielectric strength can be affected by extrinsic factors, such as the processing method, an appropriate processing method should be employed to ensure the dielectric strength of the polymer dielectric materials [45,47].

The semiconductor/dielectric interface—where the channel layer is formed—plays a crucial role in determining the charge transport characteristics of FETs. Since the device performance of 2D semiconductor FETs is sensitive to the interface, it is important to provide a defect- and impurity-free dielectric interface for 2D materials in order to enhance the device performance and operational stability of 2D semiconductor FETs [46,48,49].

In addition to the electrical characteristics, the thermal and chemical stability of polymer films needs to be ensured for the reliable operation and lithography-based down-scaling of 2D semiconductor FETs. Thus, polymer materials should be adequately designed and a proper processing method should be employed that allows them to be resistant to heat and solvent [50,51,52]. Furthermore, 2D semiconductor materials can exhibit excellent mechanical flexibility, which can be attributed to their atomically thin nature [53]. This potential applicability for flexible electronics makes polymer dielectric films more attractive due to their intrinsic mechanical softness. Therefore, the mechanical flexibility of each material constituting the device should be maximized through the material design and thickness optimization to allow applications relating to flexible 2D semiconductor FET devices [45,54].

This paper reviews recent studies that have showed improved transistor performance or new functionalities based on combinations of the aforementioned 2D semiconductors and polymer dielectrics. Polymer dielectric materials can be categorized according to their processing methods. Typically, polymers are processed in the form of films through simple and cost-effective solution-based methods, including spin-coating and spin-casting. Polymer films can also be formed by using vacuum processes, which allow for the development of high-purity dielectric films without a solvent. In addition to conventional polymer dielectrics, polymer-based materials with unique electrical properties, including ferroelectric polymers and ion gels, have also been widely investigated for 2D semiconductor devices. In Section 2, solution-processed polymer dielectric materials and 2D semiconductor FETs are introduced, with an emphasis on each representative material property and how the polymer insulating film contributes to the 2D semiconductor properties. Section 3 summarizes work on vacuum-deposited polymer dielectrics and their combination with 2D semiconductors. In Section 4 and Section 5, functional polymer dielectrics, including ferroelectric polymers and ion gels, are introduced, and their applications in relation to 2D semiconductor-based FETs are highlighted. Section 6 suggests conclusions regarding the approaches to polymer–2D semiconductor combinations and provides a summary of research efforts.

## 2. Solution-Processed Polymer Dielectric Materials for 2D Semiconductor FETs

**Figure 1:**
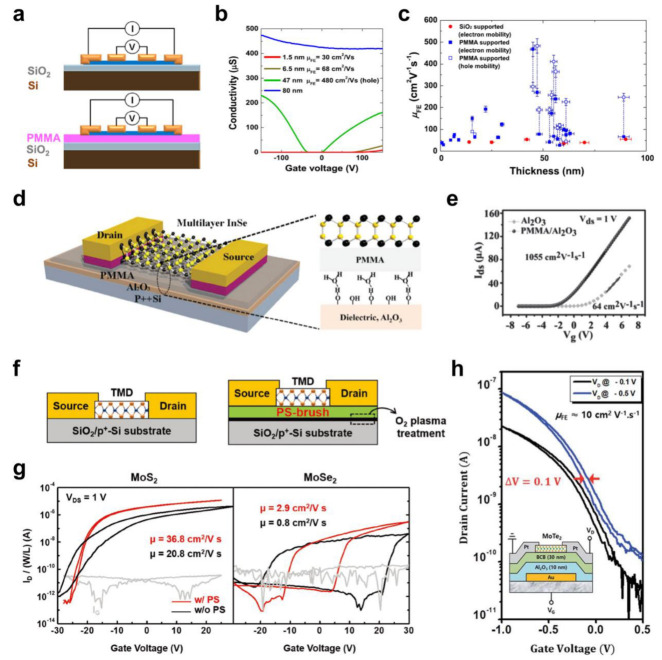
(**a**) Schematic illustration of four−probe measurement of 2D MoS_2_−based devices. (**b**) Conductivity of 2D MoS_2_ on PMMA with respect to *V*_G_. (**c**) Mobility values in relation to the thickness of the 2D MoS_2_ on PMMA compared to those for SiO_2_ [59]. Copyright © 2013, AIP Publishing. (**d**) Schematic diagram of 2D InSe FET using a PMMA/Al_2_O_3_ bilayer dielectric and (**e**) the *I*_D_−*V*_G_ characteristics compared to those without PMMA [60]. Copyright © 2013, John Wiley and Sons. (**f**) Schematic drawing of 2D TMD (MoS_2_ and MoSe_2_) FETs with SiO_2_ dielectric (left) showing PS brush interfacial layer (right) and (**g**) their transfer characteristics [61]. Copyright © 2018, John Wiley and Sons. (**h**) Transfer curves for 2D MoTe_2_ FET based on BCB/Al_2_O_3_ bilayer dielectric with different drain voltages (*V*_D_) (inset: cross−sectional device structure) [62]. Copyright © 2018, Royal Society of Chemistry.

As with various semiconductor material-based electronic devices, the interface between the semiconductor and the dielectric layer plays a vital role in determining the device performance of 2D semiconductor FETs [48,49,55,56]. Generally, abundant hydroxyl (−OH) groups and trap sites on the surfaces of inorganic dielectric materials, including conventional SiO_2_, can limit the performance of a device [57,58]. Therefore, hydrophobic polymer dielectric materials have been used to provide a favorable interface for 2D semiconductors [59,60,61,62]. Bao et al. [59] analyzed the effect of adding spin-coated poly(methyl methacrylate) (PMMA) between a 2D molybdenum disulfide (MoS_2_) semiconductor and SiO_2_ dielectric layer using a four-probe measurement, which can exclude the extrinsic factor represented by contact resistance (Figure 1a). In addition, the thickness-dependent carrier mobility (*μ*) was investigated, as shown in Figure 1b. The device, which employed 6.5 nm thick MoS_2_ on PMMA, exhibited ambipolar transport behavior, but the hole *μ* was far lower than the electron *μ* (1 and 68 cm^2^/Vs for the hole and electron, respectively). On the other hand, 47 nm thick MoS_2_ showed improved ambipolar behavior, with hole and electron *μ* values of 480 and 270 cm^2^/Vs, respectively. Moreover, regardless of the thickness, 2D MoS_2_ layers on PMMA commonly exhibited enhanced *μ* compared to those on SiO_2_ (Figure 1c). In addition, ambipolar transport behavior could only be observed with PMMA, which verified that the charge transport characteristics of 2D semiconductors could be improved with polymeric layers.

The interfacial optimization of 2D semiconductors with an interfacial PMMA layer has been demonstrated using FET devices. Feng et al. [60] fabricated FET devices based on 2D indium selenide (InSe) that exhibit high mobility because of the light effective mass of electrons. A PMMA/Al_2_O_3_ bilayer dielectric was employed where high-*k* Al_2_O_3_ enabled low-voltage operation (<8 V), and the surface of the inorganic dielectric was modified by introducing a PMMA layer (Figure 1d). Compared to the FET devices with a single-layer Al_2_O_3_ dielectric, those with a bilayer dielectric showed far higher *μ* of up to 1055 cm^2^/Vs, which was attributed to the suppression of charge carrier scattering caused by Coulomb impurities from hydroxyl groups at the surface of the oxide dielectric, as shown in the *I*_D_ − *V*_G_ relationship (Figure 1e).

In addition to PMMA, various polymer materials can be used to improve 2D semiconductor FET devices. For example, Jeong et al. [61] introduced a polystyrene (PS) brush interfacial layer on top of SiO_2_ using spin-coating, as shown in Figure 1f. FETs with two different 2D TMD semiconductors (MoS_2_ and MoSe_2_) were fabricated, and both exhibited improved *μ* values with the PS brush, with considerably reduced hysteresis in the transfer curves (Figure 1g). In addition, low-voltage operation of these FETs was achieved by replacing the SiO_2_ with Al_2_O_3_. By further improving FET performance using an Al_2_O_3_ encapsulation layer (*μ* = ~11.2 cm^2^/Vs), a piezoelectric touch sensor with an organic light-emitting diode (OLED) indicator was produced, and the 2D MoSe_2_ FET-integrated touch sensor showed more distinguishable on/off states because of the lower *V*_T_, despite the relatively lower *μ*.

Divinyltetramethyldisiloxane-bis(benzocyclobutene) (BCB) is another example of an interfacial polymeric layer that has been frequently used to enhance organic FET (OFET) performance [63,64]. Yoon et al. [62] fabricated 2D MoS_2_ and MoTe_2_ FETs with a spin-casted BCB interfacial layer, and the hysteresis was reduced significantly thanks to the absence of a hydroxyl group in the BCB layer. Using high-*k* Al_2_O_3_, 1 V operation of the 2D MoTe_2_ FET was demonstrated with high *μ* (~10 cm^2^/Vs) and minimized hysteresis, as shown in Figure 1h.

**Figure 2:**
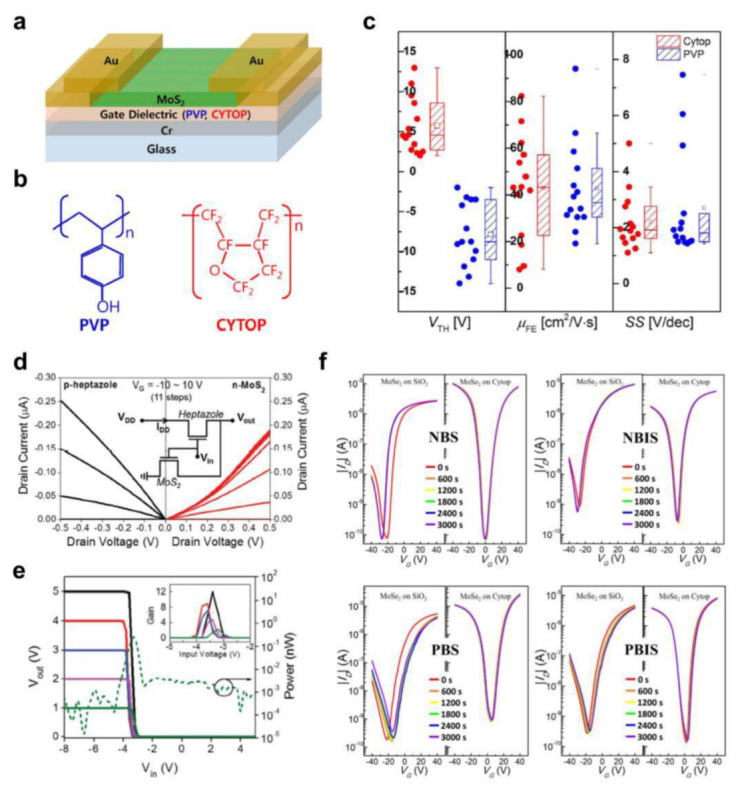
(**a**) Device structure for 2D MoS_2_ FETs based on polymer dielectric materials and (**b**) the chemical structures of PVP and CYTOP. (**c**) The *V*_T_, *μ*, and *SS* distributions for 14 2D MoS_2_ FET devices [68]. Copyright © 2016, AIP Publishing. (**d**) The output curves of the p−type heptazole OFET and n−type 2D MoS_2_ FET, which both utilized a CYTOP/Al_2_O_3_ bilayer dielectric, and (**e**) the VTC curves of the resulting hybrid CMOS inverter according to the *V*_DD_ (inset: voltage gain profile) [39]. Copyright © 2015, John Wiley and Sons. (**f**) Changes in the transfer curves in the NBS (top left), NBIS (top right), PBS (bottom left), and PBIS (bottom right) measurements for 2D MoSe_2_ FETs with a CYTOP interfacial layer compared to those without CYTOP [69]. Copyright © 2021, Elsevier.

Employing 2D semiconductor FETs for practical applications requires improvements in both *μ* and *V*_T_ [65,66]. For example, 2D MoS_2_ FETs have generally shown *V*_T_ in the negative *V*_G_ range (depletion mode), which may increase power consumption [67]. In this context, CYTOP has been investigated as a gate dielectric layer for 2D TMD FETs [39,68,69] due to its electron-withdrawing property resulting from the fluorocarbon chains, as well as its excellent insulating performance [70,71]. Yoo et al. [68] utilized CYTOP as a gate dielectric in 2D MoS_2_ FETs and compared them with a poly(4-vinylphenol) (PVP) dielectric (Figure 2a,b). Both polymer dielectric layers could be spin-coated on top of a gate electrode without an additional dielectric layer thanks to the outstanding dielectric properties [71,72,73]. As shown in the statistical analysis in Figure 2c, *V*_T_-adjusting effects were observed for the CYTOP gate dielectric. The 2D MoS_2_ FETs with PVP showed a negative *V*_T_ of −14 V (depletion mode). In contrast, those with CYTOP exhibited *V*_T_ = 4.6 V, enabling the device to be turned off at 0 V (enhancement mode), which was attributed to the strong electron-withdrawing capacity of the fluoroalkyl chain in CYTOP. Moreover, the other device parameters, including *μ* (~40 cm^2^/Vs) and the subthreshold swing (*SS*) (~2.5 V/dec.), were kept at similar levels (Figure 2c).

Lee et al. [39] also reported *V*_T_ controllability using CYTOP. They introduced a thin (~30 nm) CYTOP interfacial layer using spin-coating to reduce the *V*_T_ of 2D MoS_2_ FETs. In addition, Al_2_O_3_ deposited using ALD was employed to ensure low-voltage operation (<10 V). In this work, 2D MoS_2_ semiconductors with two different thicknesses (3 layers (3L) and 10 layers (10L)) were used to fabricate FET devices, and they both exhibited a reduced *V*_T_ with CYTOP (from −12.9 V to −3.2 V for the 3L MoS_2_ FETs and from −25.5 V to −5.7 V for the 10L MoS_2_ FETs). The *μ* values of the 3L MoS_2_ FETs were practically unchanged (6.2 cm^2^/Vs without CYTOP and 6.0 cm^2^/Vs with CYTOP) and the 10L MoS_2_ FETs showed slightly improved *μ* with CYTOP (12.7 cm^2^/Vs without CYTOP and 14.9 cm^2^/Vs with CYTOP). Both devices also exhibited improved *SS* with the addition of CYTOP. The 3L MoS_2_ FET with CYTOP exhibited more significantly reduced *V*_T_, so this FET was integrated with a p-type heptazole organic FET (OFET) to produce a complementary metal–oxide–semiconductor (CMOS) inverter with a vertical structure, with both FETs exhibiting balanced electrical characteristics (Figure 2d). The resulting hybrid CMOS inverter showed full-swing operation, with a maximum voltage gain of ~12 at a supply voltage (*V*_DD_) of 5 V (Figure 2e). In addition, pixel operation of the CMOS inverter was demonstrated by exploiting the photo-responsive property of the 2D MoS_2_, and the voltage transfer curve (VTC) was shifted toward the negative voltage direction with red and green light illumination.

In addition to the electron-withdrawing property, the fluoroalkyl chain in CYTOP has been found to result in substantial hydrophobicity and minimal charge trapping, enhancing the stability of FETs [74,75,76]. Hong et al. [69] introduced a CYTOP interfacial layer to 2D MoSe_2_ FETs. Compared to those without the CYTOP layer, the 2D MoSe_2_ FETs with the CYTOP interfacial layer showed a *V*_T_ shift toward the positive voltage direction, leading to balanced ambipolar behavior. While the 2D MoSe_2_ FETs without CYTOP exhibited hole and electron *μ* values of 0.3 and 25.5 cm^2^/Vs, respectively, those with CYTOP showed hole and electron *μ* values of 18.4 and 16.4 cm^2^/Vs, respectively. Moreover, the stability of the 2D MoSe_2_ FETs was enhanced in negative/positive bias stability (NBS/PBS) and negative/positive bias illumination stability (NBIS/PBIS) measurements after introducing the CYTOP interfacial layer due to the minimized charge trapping, as shown in Figure 2f. In addition, when using two MoSe_2_ FETs with well-balanced ambipolar transport characteristics, an inverter was demonstrated with which full-swing operation with a maximum gain of ~9.7 and high stability for up to 5000 cycles was achieved.

In addition to their role as gate dielectric layers, the polymer materials containing electron-withdrawing and electron-donating functionalities, such as CYTOP and polyethyleneimine (PEI), have also been used as an additional layer to systematically optimize the electrical characteristics of 2D FETs [77,78,79,80,81,82]. Furthermore, hydrophobic polymers have been used as barrier layers to improve the environmental stability of ambient-instable 2D materials, such as black phosphorus (BP) and nanostructured 2D materials [83,84,85,86,87,88].

**Figure 3:**
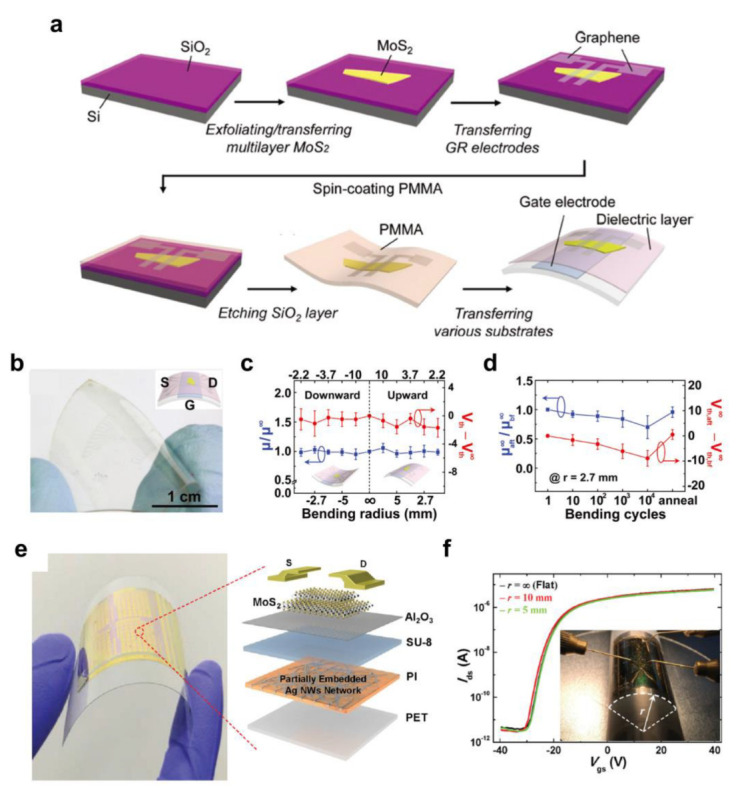
(**a**) Schematic diagram of the fabrication process and (**b**) photograph of the flexible and transparent 2D MoS_2_ FET with graphene S/D electrode and ITO gate electrode on PET substrate. The change in *μ* and *V*_T_ with respect to the (**c**) bending radius and (**d**) bending cycles [93]. Copyright © 2013, John Wiley and Sons. (**e**) Photograph (left) and schematic illustration of the device structures of the flexible 2D MoS_2_ FETs with an Al_2_O_3_/SU−8 bilayer dielectric and AgNW gate electrode. (**f**) Change in the transfer curves of the flexible 2D MoS_2_ FETs in relation to the bending radius [21]. Copyright © 2016, John Wiley and Sons.

One of the important advantages of polymer dielectric materials is their mechanical flexibility, which enables flexible FET devices to be combined with an atomically 2D semiconductor. On the other hand, the mechanically exfoliated 2D semiconductors require a photolithographic micropatterning process [89,90], and chemical vapor deposition (CVD)-based large-area synthesis methods demand high-temperature and designated surfaces [91,92], which may be incompatible with flexible substrates. Therefore, an appropriate fabrication procedure should be developed that includes transferring 2D materials into flexible substrates to implement flexible FETs. Yoon et al. [93] demonstrated flexible 2D MoS_2_ FETs on a polyethylene terephthalate (PET) substrate. Moreover, graphene was used for the source/drain (S/D) electrodes to achieve optical transparency and low contact resistance. As shown in Figure 3a, mechanically exfoliated MoS_2_ was placed on a SiO_2_/Si substrate, which was followed by the transfer of patterned graphene S/D electrodes. After coating a PMMA supporting layer and subsequent chemical etching of SiO_2_, 2D MoS_2_ and graphene S/D electrodes could be transferred into the flexible substrate containing a gate electrode and dielectric layer. Indium tin oxide (ITO) was used as a gate to implement transparent FETs and crosslinked PVP (c-PVP) formed using spin-coating was employed due to its excellent dielectric properties [72,73]. The resulting FETs exhibited optical transmittance of 74%, which is highly transparent considering the optical transmittance of bare PET substrate (~86%) (Figure 2c). Moreover, the flexible FET devices maintained their electrical characteristics (*μ*~4.7 cm^2^/Vs and *I*_on_/*I*_off_ higher than 10^4^) under a bending radius of up to 2.2 mm and repeated bending for up to 10,000 cycles with a fixed bending radius (2.7 mm) (Figure 3c,d).

Song et al. [21] reported the development of flexible 2D MoS_2_ FETs on spin-coated polyimide (PI) substrate. As a gate electrode, a silver nanowire (AgNW) network was deposited and laser-welded, after which the PI was spin-coated. The AgNW network-embedded PI layer was detached and attached mechanically to the carrier substrate. In this work, an Al_2_O_3_/SU-8 bilayer dielectric was used where the SU-8 was spin-coated to ensure coverage of the AgNW network, and then Al_2_O_3_ was applied through ALD (Figure 3e). This bilayer dielectric structure was found to be beneficial for securing the proper insulating properties and producing high-performance 2D MoS_2_ FETs. The MoS_2_ was mechanically exfoliated, and the S/D electrode was fabricated using conventional photolithography. A flexible FET device was completed by detaching the PI layer from the carrier substrate and attaching it to a flexible PET substrate. The resulting flexible 2D MoS_2_ FETs exhibited high performance (*μ*~141.3 cm^2^/Vs) and excellent mechanical flexibility. There was no significant change in the transfer curves, and the *μ* change was less than 9% even with a bending radius of 5 mm (Figure 3f). Moreover, although the transfer curve was shifted slightly toward a higher voltage direction, the *I*_on_/*I*_off_ was preserved at ~10^5^ with a *μ* change of less than 10% and a *V*_T_ change of less than 6 V under repeated bending with 1000 cycles.

The previous research described above strongly suggests that polymer materials can optimize the performance of FETs based on 2D materials by providing a favorable interface for charge transport and chemical functionalities. Table 1 lists the electrical characteristics of the 2D semiconductor FETs with solution-processed polymer gate dielectrics. In addition, with a proper process scheme, flexible 2D semiconductor-based FETs can be implemented, maximizing the advantageous mechanical properties of polymer materials (i.e., mechanical flexibility).

## 3. Vacuum-Deposited Polymer Dielectric Materials for 2D Semiconductor FETs

**Figure 4:**
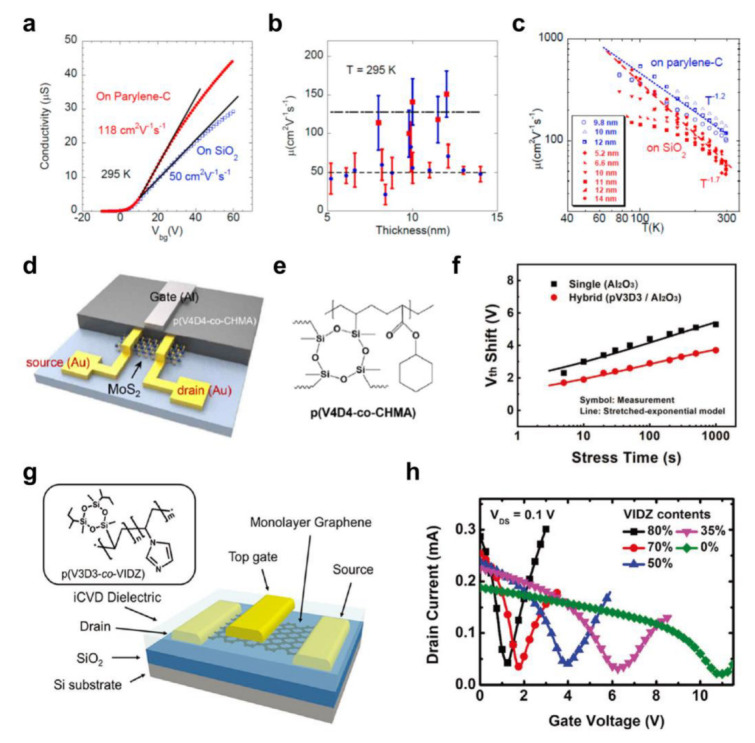
(**a**) Conductance−*V*_G_ curves, (**b**) *μ* in relation to the MoS_2_ thickness, and (**c**) the change in *μ* in relation to the temperature for 2D MoS_2_ FETs fabricated with a parylene interfacial layer compared to those without a parylene layer (on SiO_2_) [96]. Copyright © 2014, American Chemical Society. (**d**) Schematic illustration of the top−gate, 2D MoS_2_ FET based on the iCVD dielectric and (**e**) the chemical structure of P(V4D4−co−CHMA) [106]. Copyright © 2018, American Chemical Society. (**f**) The *V*_T_ shift with the stress time for the 2D MoS_2_ FETs with the hybrid dielectric (PV3D3/Al_2_O_3_) compared to those without the PV3D3 dielectric in NBIS measurements [107]. Copyright © 2021, John Wiley and Sons. (**g**) Schematic diagram of the device structure for a graphene FET with iCVD-based P(V3D3−co−VIDZ). (**h**) Change in the *I*_D_−*V*_G_ curves with the VIDZ contents in P(V3D3−co−VIDZ) dielectric layers [108]. Copyright © 2018, John Wiley and Sons.

Although the process may become somewhat complicated due to the need for equipment, such as a vacuum chamber and a vacuum pump, vacuum-deposited polymer dielectric materials have distinct advantages compared to conventional solution-based processes [94]. For example, high-purity polymer thin films can be obtained due to the absence of solvents and additives; thus, the potential degradation of FET performance caused by residual impurities can be eliminated. The representative polymer dielectric material processed using vacuum deposition is parylene, which can be deposited using CVD and has been frequently used to optimize the performance of 2D semiconductor FETs based on graphene and TMDs [95,96,97,98,99]. Chamlagain et al. [96] used a parylene interfacial layer in 2D MoSe_2_ FETs on a SiO_2_/Si substrate, which exhibited significantly improved *μ* (~118 cm^2^/Vs) compared to those without a parylene layer (~50 cm^2^/Vs), as shown in Figure 4a. Moreover, in the four-probe measurement, the 2D MoSe_2_ showed higher mobility with the parylene interfacial layer with different 2D MoSe_2_ thicknesses (Figure 4b). The authors performed temperature-dependent measurements to shed light to the origin of the improved *μ* with the parylene layer. The MoSe_2_ FETs followed the rule *μ* ≈ *T*^−γ^ regardless of the presence of a parylene interfacial layer, meaning there were limitations due to phonon scattering in both cases (Figure 4c). However, the *γ* value decreased with the use of parylene (*γ*~1.7 on SiO_2_ and *γ*~1.2 on parylene). The authors deduced that the additional polar optical phonon scattering at the SiO_2_ surface could be reduced with the parylene interfacial layer, which improved the *μ* of the 2D MoSe_2_ FETs.

Recently, initiated chemical vapor deposition (iCVD) has become a useful tool for synthesizing high-purity polymer dielectric films [47,100]. Unlike parylene CVD, various polymer films can be deposited with iCVD [38,101,102]. In addition, copolymer films with diverse combinations can be formed because mixing between arbitrary monomers is not constrained in the gas phase [103,104,105]. Moreover, this process is based on physical adsorption, which makes it possible to form a uniform, ultrathin (a few tens of nanometers) polymer dielectric film on top of dangling bond-free 2D semiconductors [47,94]. Therefore, by using a low processing temperature, iCVD-based polymer dielectrics enabled the fabrication of 2D semiconductor FETs with a top-gate geometry [40,106,107,108,109].

Kim et al. [106] synthesized poly(2,4,6,8-tetramethyl-2,4,6,8-tetravinylcyclotetrasiloxane-co-cyclohexyl methacrylate) (P(V4D4-co-CHMA)) using iCVD (Figure 4d,e). The resulting copolymer dielectric exhibited robust insulating performance even with a ~40 nm thickness, and the breakdown electric field (*E*_break_) was >3 MV/cm. Low-voltage (<5 V), top-gate 2D MoS_2_ FETs were fabricated using the copolymer dielectric. The resulting FETs showed higher *μ* (~35.1 cm^2^/Vs) and lower *SS* (~0.2 V/dec.) compared to those based on the SiO_2_ dielectric with a bottom-gate geometry (*μ =* ~19.2 cm^2^/Vs and *SS =* ~1.6 V/dec.). The temperature-dependent measurements showed that phonon-mode quenching occurred with the copolymer dielectric, resulting in an improvement in carrier transport. In addition, with the reduced interface charge trap density and top-gate geometry, the 2D MoS_2_ FETs with P(V4D4-co-CHMA) showed enhanced operational and environmental stability. Similar results were reported by Park et al. [107], who deposited poly(1,3,5-trimethyl-1,3,5-trivinyl cyclotrisiloxane) (PV3D3) via iCVD between the 2D MoS_2_ and Al_2_O_3_. An improved *μ* value was obtained in the 2D MoS_2_ FETs containing the interfacial PV3D3 (~10.4 cm^2^/Vs) with reduced hysteresis in the transfer curve compared to those without PV3D3 (~6.3 cm^2^/Vs). Moreover, the *V*_T_ shift was reduced from −5.3 V to −3.7 V in the NBIS stability measurement by utilizing the interfacial PV3D3 layer (30% reduction), as shown in Figure 4f. These results show that the interface trap density can be reduced with iCVD-based polymer dielectrics, leading to improved charge transport and stability for 2D semiconductor FETs.

As mentioned above, copolymer dielectric materials based on various combinations of different monomers can be designed and synthesized via the iCVD process, which enables systematic optimization of the FET performance. Oh et al. [108] synthesized poly(1,3,5-trimethyl-1,3,5-trivinyl cyclotrisiloxane-co-1-vinylimidzole) (P(V3D3-co-VIDZ)) dielectric films and utilized them as a gate dielectric layer in graphene transistors with a top-gate geometry (Figure 4g). The copolymer dielectric materials exhibited robust insulating performance even with 20 nm thickness regardless of the chemical composition owing to the solvent-free nature of the iCVD process. Furthermore, the copolymer films could induce a negative *V*_T_ shift, which resulted from the electron-donating imidazole moiety. As shown in Figure 4h, the Dirac voltage (*V*_Dirac_), which represents the minimum conductance of the graphene, decreased as the 1-vinylimidzole (VIDZ) contents were increased. The *V*_Dirac_ was 11 V with 0% VIDZ moiety (PV3D3 homopolymer), but it was shifted to approximately 1 V with 80% VIDZ moiety, and the hole and electron values were enhanced to 7270 and 3860 cm^2^/Vs, respectively. Using this high-performance graphene FET based on polymer dielectrics, the electrical characteristics of the flexible RF device barely changed with the applied tensile strain of up to 1.3%. Table 1 lists the electrical characteristics of 2D FETs based on 2D semiconductors and vacuum-deposited polymer gate dielectrics.

With the increasing demand for data storage as big data emerges, the importance of developing nonvolatile flash memory capable of storing information is also increasing [110,111]. Therefore, various 2D semiconductor FET-based flash memory devices have been demonstrated [112,113,114]. In flash memory devices, charges can be stored in a floating gate (FG) by applying *V*_G_ (typically in the form of a pulse), resulting in different programming/erasing states [115]. The applied *V*_G_ is divided into the blocking dielectric layer (BDL) and tunneling dielectric layer (TDL) according to the gate-coupling ratio, which is proportional to the dielectric constant of the BDL and inversely proportional to the dielectric constant of the TDL [115,116]. Therefore, a low-*k* dielectric material in the TDL is vital for enabling low-voltage operation of flash memory devices. In addition, the thickness of the TDL should be kept low because charge trapping is based on a tunneling mechanism. From this point of view, polymer materials may be promising candidates for TDLs in 2D semiconductor-based nonvolatile flash memories.

**Figure 5:**
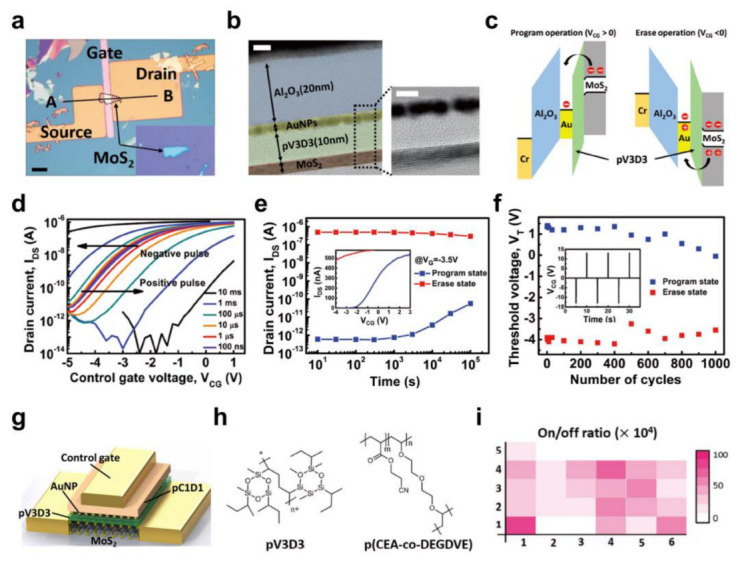
(**a**) Optical microscopy (scale bar: 10 μm) and (**b**) cross−sectional transmission electron microscopy (TEM) images (scale bar: 5 nm) of the 2D MoS_2_ flash memory. (**c**) Electronic band diagrams for programming and erasing operations. (**d**) The change in the transfer curves in relation to the positive/negative pulse width (*V*_G_ = ±13 V), (**e**) the change in *I*_D_ with respect to time, and (**f**) the change in *V*_T_ monitored in repetitive programming/erasing cycles [40]. Copyright © 2017, John Wiley and Sons. (**g**) Schematic illustration of the 2D MoS_2_ flash memory based on the polymer dielectric films and (**h**) chemical structures of the polymer materials. (**i**) The *I*_on_/*I*_off_ distribution of the fabricated C−memory devices [109]. Copyright © 2019, John Wiley and Sons.

Woo et al. [40] fabricated a nonvolatile flash memory device using a 2D MoS_2_ semiconductor with a top-gate geometry (Figure 5a). iCVD-based PV3D3 was used as a TDL because of its sufficiently low dielectric constant (~2.2) and excellent insulating performance, even at a thicknesses of a few nanometers [47]. As shown in Figure 5b, the ultrathin (~10 nm) PV3D3 TDL uniformly covered the dangling bond-free, 2D MoS_2_ surface resulting from the physical adsorption and surface-growing deposition mechanism of the iCVD, which is difficult to achieve with the ALD process. In addition, high-*k* Al_2_O_3_ was used as a BDL, which enabled further reductions in the programming/erasing voltage of the flash memory device (Figure 5c). Gold nanoparticles (AuNPs) were utilized for the FG. Due to the low thickness of the dielectric layers, low-voltage operation (~6 V) could be achieved. The systematic *V*_T_ shift was obtained with programming and erasing operations, leading to the large memory window (5.2 V) (Figure 5d). Furthermore, the fabricated flash memory device exhibited highly reliable operation. As shown in Figure 5e, both states were well-maintained even at 10^5^ s after programming/erasing operations, indicating good retention characteristics. In addition, the memory states were preserved without degradation of the device performance over 10^3^ cycles of repetitive operations (Figure 5f).

Yang et al. [109] reported the development of a 2D MoS_2_ nonvolatile flash memory device where all the dielectric layers were composed of polymer materials, expanding flash memory into flexible electronics. As shown in Figure 5g,h, poly(2-cyanoethyl acrylate-co-diethylene glycol divinyl ether) (P(CEA-co-DEGDVE)) with the optimal chemical composition (named PC1D1) was introduced as a BDL and exhibited a dielectric constant greater than 6 and robust insulating properties [38,102,117]. Moreover, in addition to the mechanically exfoliated MoS_2_ (E-memory), a few-layer MoS_2_ synthesized via the CVD process was used as a semiconductor for the flash memory (C-memory). By exploiting these large-area processable techniques (CVD for MoS_2_ and iCVD for polymer films), a uniform distribution of the electrical characteristics of the C-memory was achieved. As shown in Figure 5i, the *I*_on_/*I*_off_ was higher than 10^4^ for all the working C-memory devices. Some non-working devices are present (marked as white regions) because the MoS_2_ layer was damaged during transfer rather than because of the non-uniform electrical characteristics of CVD-grown materials. Thus, the device-to-device uniformity can be further improved by optimizing the transfer process. Nevertheless, the C-memory showed a large memory window and good retention characteristics and cyclic endurance, making it comparable to E-memory.

## 4. Ferroelectric Polymers for 2D Semiconductor FETs

**Figure 6:**
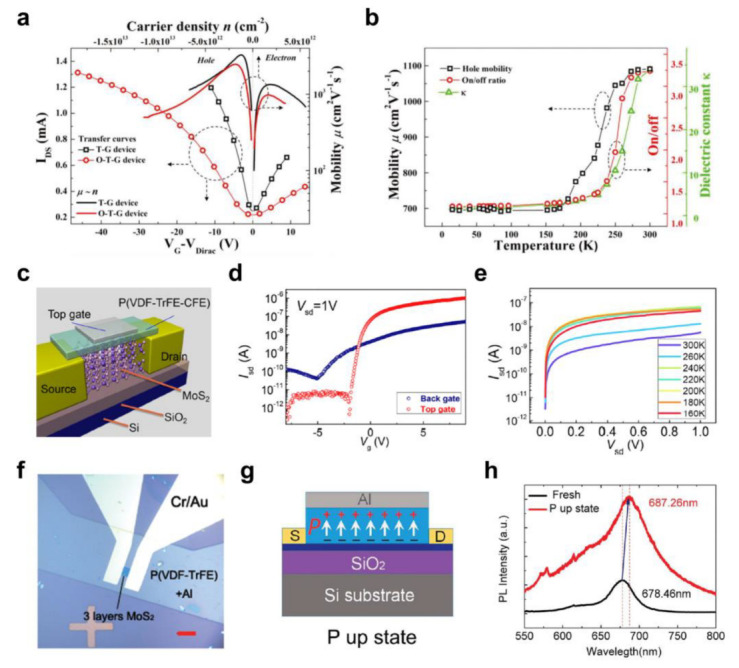
(**a**) *I*_D_ vs. *V*_G_ −*V*_Dirac_ transfer curves for the graphene FETs with the P(VDF−TrFE−CFE) single−layer dielectric (T–G device) and with the P(VDF−TrFE−CFE)/SiO_2_ bilayer dielectric (O–T–G device). (**b**) The change in the *μ* and *I*_on_/*I*_off_ for the graphene T–G device, and the dielectric constant of the P(VDF−TrFE−CFE) in relation to the temperature [124]. Copyright © 2020, John Wiley and Sons. (**c**) Schematic illustration of 2D MoS_2_ FETs with the P(VDF−TrFE−CFE) dielectric layer fabricated on SiO_2_/Si substrate and (**d**) their transfer characteristics. (**e**) Change in output characteristics in relation to the temperature for the top−gate 2D MoS_2_ FETs with the P(VDF−TrFE−CFE) dielectric layer [125]. Copyright © 2016, American Chemical Society. (**f**) Optical microscope image of the photodetector device containing three−layered MoS_2_ and P(VDF−TrFE) and (**g**) the schematic diagram of the device with negative *V*_G_ applied (P up state). (**h**) The PL spectrum of 2D MoS_2_ in P up state compared to that without an external electric field (fresh) [126]. Copyright © 2015, John Wiley and Sons.

Polyvinylidene fluoride (PVDF)-based ferroelectric polymers, such as poly(vinylidene fluoride-trifluoroethylene) (P(VDF-TrFE)) and poly(vinylidene fluoride-trifluoroethylene-chlorofluoroethylene) (P(VDF-TrFE-CFE)) are attractive materials for low-voltage operation of FETs owing to their high dielectric constants and excellent dielectric properties [118,119,120]. Ferroelectric polymers have hence been studied extensively for 2D semiconductor FET and photodetector applications [121,122,123,124,125,126]. Wen et al. [124] employed a P(VDF-TrFE-CFE) dielectric layer to fabricate graphene transistors. Before applying the ferroelectric polymer to the FET devices, the annealing temperature was optimized by measuring the change in the dielectric constant and surface morphology of the P(VDF-TrFE-CFE) at four different temperatures (80, 100, 120, and 140 °C). The dielectric constant increased continuously with a concomitant decrease in the surface roughness of the film as the annealing temperature was increased to 120 °C. At 140 °C, however, the dielectric loss increased significantly with the smallest dielectric constant, indicating film degradation. Thus, the annealing temperature was optimized to 120 °C. Graphene transistors were fabricated with P(VDF-TrFE-CFE) based on two different structures: one utilized P(VDF-TrFE-CFE) with a SiO_2_ dielectric layer (O–T–G device) and the other used a single P(VDF-TrFE-CFE) layer (T-G device). As shown in Figure 6a, the T–G device showed better performance, with hole and electron *μ* values of 3170 and 1630 cm^2^/Vs, respectively, and lower operating voltage (~10 V). This enhanced performance was attributed to the stronger screening effect of the Coulomb scattering in the T–G device because the dielectric constant of the single layer P(VDF-TrFE-CFE) (~33.6) was higher than that of the SiO_2_-P(VDF-TrFE-CFE) composite (~13.5). Moreover, the device performance correlated directly with the dielectric constant, which was proportional to the temperature, verifying the advantage of using high-*k* dielectric materials in 2D semiconductor FETs (Figure 6b).

Similar experimental results were observed with 2D TMD FETs. Chen et al. [125] fabricated a 2D MoS_2_ FET using a P(VDF-TrFE-CFE) dielectric layer on a SiO_2_/Si substrate with a top-gate geometry (Figure 6c). The top-gate FET showed higher *μ* (51.9 cm^2^/Vs) compared to the bottom-gate FET with a SiO_2_ dielectric layer (3.5 cm^2^/Vs), as well as an enhanced *I*_on_/*I*_off_ ratio (Figure 6d). This superior device performance could be attributable to the screening effect of the Coulomb scattering caused by the high dielectric constant of the P(VDF-TrFE-CFE), which would be consistent with the results above. On the other hand, an unusual temperature dependency was observed for the *I*_D_−drain voltage (*V*_D_) output characteristics where *I*_D_ increased at lower temperatures. This was attributed to the unique characteristics of the ferroelectric polymer, where an additional gating effect occurs with the remnant polarization of the P(VDF-TrFE-CFE), which became prominent with decreasing temperature.

The stable remnant polarization and high dielectric constant of ferroelectric polymers make them suitable for fabricating high-performance photodetectors because a strong local electric field can induce a fully depleted state in the semiconductor channel, which can enhance the sensitivity. Wang et al. [126] reported a photodetector based on 2D MoS_2_ and ferroelectric P(VDF-TrFE) (Figure 6f), which showed a high electron *μ* of 86.5 cm^2^/Vs. In the fully depleted state, where the negative *V*_G_ was applied (P up state in Figure 6g), the photo-generated current dominated the channel current. Highly sensitive operation of the photodetector could be achieved with responsivity of up to 2570 A/W and detectivity of ~2.2 × 10^12^ Jones. In addition, a rapid photo-response (~2 ms) was achieved, which may have been due to the passivated interface between the 2D MoS_2_ and ferroelectric polymer resulting from the fluorine or hydrogen atoms in P(VDF-TrFE). Moreover, a light wavelength longer than the wavelength that fresh 2D MoS_2_ can respond to was detected. This was because the band structure of 2D MoS_2_ can be adjusted with an external electric field, as observed in the photoluminescence measurements (Figure 6h). This tunable band structure achieved in the 2D MoS_2_ by applying an electric field through a ferroelectric polymer was also confirmed in the following study [125].

**Figure 7:**
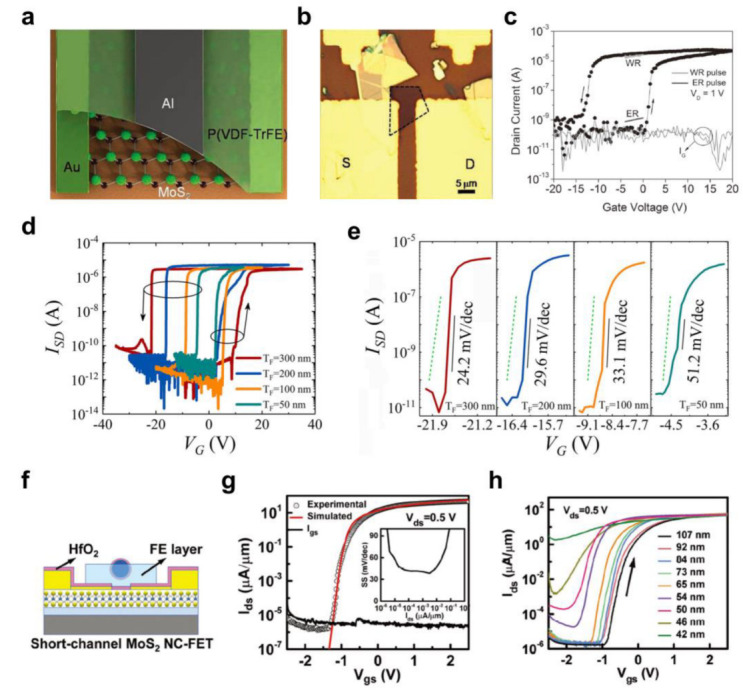
(**a**) Schematic illustration and (**b**) optical microscope image of the 2D MoS_2_ memory device using ferroelectric P(VDF−TrFE). (**c**) The transfer characteristics of the ferroelectric memory with single−layer MoS_2_ [127]. Copyright © 2012, John Wiley and Sons. The transfer curves of 2D MoS_2_ NC−FETs based on P(VDF−TrFE) in the (**d**) overall operating voltage range and (**e**) subthreshold region [133]. Copyright © 2017, Springer Nature. (**f**) Schematic illustration of the short−channel 2D MoS_2_ NC−FETs employing HfO_2_/P(VDF−TrFE). (**g**) The experimentally measured transfer curve compared to that obtained in the numerical simulation (inset: the extracted *SS* at each *I*_D_ level). (**h**) The change in transfer curves in relation to the channel length [134]. Copyright © 2018, John Wiley and Sons.

As described above, stable remnant polarization is one of the attractive electrical characteristics of ferroelectric polymers. Hence, ferroelectric memory devices using 2D semiconductors and ferroelectric polymer dielectrics have been developed [122,127,128,129]. Lee et al. [127] reported the development of nonvolatile ferroelectric memory based on 2D MoS_2_ using P(VDF-TrFE), as shown in Figure 7a,b. With a single-layer MoS_2_, a large memory hysteresis window (~14 V) was obtained with a *μ* of ~220 cm^2^/Vs (Figure 7c). Stable operation was achieved, and the two distinctive (programming/erasing) states were maintained for 1000 s without noticeable *I*_D_ variations. Moreover, those distinctive states were preserved with repetitive operations (~10 programming/erasing cycles). On the other hand, the memory window was reduced significantly from ~14 V to ~6 V when the channel layer was changed to double-layer MoS_2_, and *SS* was increased from ~300 mV/dec. to ~2 V/dec. The memory window was decreased further to ~3 V with triple-layer MoS_2_. This shortened memory window might have been due to the depolarization effects and the degraded *SS* behavior. Nevertheless, good retention characteristics and cycling endurance were achieved in the double-layer MoS_2_ device, which verified that reliable memory operation could be achieved using ferroelectric polymers.

Further interesting behavior that can be achieved using ferroelectric polymers is the negative capacitance (NC) effect, which can overcome the theoretical lower limit of *SS* (~60 mV/dec.), leading to the steep switching of FETs [130,131]. NC-FETs with 2D semiconductors have been produced by using ferroelectric polymers [132,133,134]. Wang et al. [133] developed NC-FETs based on 2D MoS_2_ and ferroelectric polymer. P(VDF-TrFE) with four different thicknesses was utilized (50, 100, 200, and 300 nm). Counter-clockwise hysteresis in the transfer curves was observed for all FET devices (Figure 7d). The hysteresis window widened with the increase in the thickness of the P(VDF-TrFE) because the coercive voltage is strongly related to the thickness of a ferroelectric polymer. Moreover, all the FETs exhibited *SS* < 60 mV/dec. (Figure 7e). The measured *SS* values for P(VDF-TrFE) thicknesses of 300, 200, 100, and 50 nm were 24.2, 29.6, 33,1, and 51.2 mV/dec., respectively, which showed a clear correlation between the *SS* value and the thickness of the ferroelectric polymer layer. This observation was attributed to the technical limitations, as a “dead” layer in the P(VDF-TrFE) at the interface for the top-gate electrode was confirmed using transmission electron microscopy (TEM) analysis. *SS* increased as the P(VDF-TrFE) thickness decreased because a certain portion of the ferroelectric polymer could not contribute to the NC effect.

Liu et al. [134] fabricated NC-FETs using 2D MoS_2_ and a HfO_2_/P(VDF-TrFE) bilayer dielectric (Figure 7f). The HfO_2_ layer was inserted between the 2D MoS_2_ and P(VDF-TrFE) to achieve a stable demonstration of the NC effect. Moreover, silver nanowires were employed as the gate electrode to produce short-channel (<100 nm) NC-FETs. The thicknesses of the HfO_2_ and P(VDF-TrFE) were optimized to 4 and 26 nm, respectively. The resulting devices exhibited electrical characteristics that perfectly matched with the numerical simulation, with low operating voltage (<3 V) and low gate leakage current (*I*_G_) (Figure 7g). In particular, *SS* < 60 mV/dec. was obtained in ~4 orders of the *I*_D_ range. Figure 7h shows the transfer characteristics of the 2D MoS_2_ NC-FETs, with the channel length ranging from 42 to 130 nm. When the channel length shrank, degradation in the performance of the device was observed. Nevertheless, stable operation of the NC-FETs was achieved with a low SS (<60 mV/dec.) and high transconductance when the channel length was longer than 60 nm. The subthreshold characteristics were improved further by lowering the temperature through the enhanced polarization of the P(VDF-TrFE), and the steep switching performance was fully preserved even in NBS and PBS conditions. Table 2 lists the electrical characteristics of 2D semiconductor FETs with ferroelectric polymer dielectric materials.

## 5. Ion Gels and Polymer Electrolytes for 2D Semiconductor FETs

Low-voltage 2D semiconductor FETs have been produced based on ion gels and polymer electrolytes, taking advantage of their high capacitance resulting from an electrical double layer (EDL) [135,136,137,138,139,140,141,142,143]. For example, the electrical characteristics of the 2D semiconductors newly synthesized using CVD could have been investigated using ion gels [144,145]. In addition, the contact resistance can be reduced with polymer electrolytes and ionic liquids by lowering the sheet resistance of 2D materials [146,147]. Generally, ion gels are produced by dissolving polymers in ionic liquids, such as 1-ethyl-3-methylimidazolium bis(trifluoromethylsulfonyl)imide ([EMIM][TFSI]), and polymer electrolytes are prepared from mixtures of polymers and electrolytes, such as LiClO_4_, which means that the unique properties—including EDL formation—are due to the ionic liquids or electrolytes rather than the polymer matrices [148,149]. Therefore, this review briefly introduces some ion gels and polyelectrolytes for 2D semiconductor FETs.

**Figure 8:**
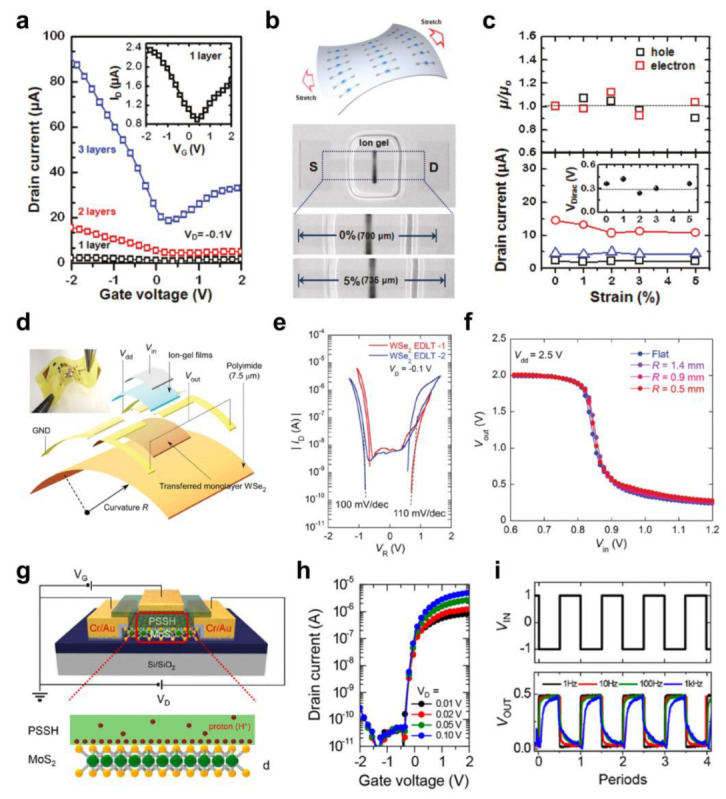
(**a**) The transfer curves of the graphene FETs employing [EMIM][TFSI]/PS−PMMA−PS ion gels in relation to the thickness of graphene. (**b**) Schematic illustration (top) and photography (bottom) of the stretching test. (**c**) The changes in the *μ*, *I*_D_, and *V*_Dirac_ of the graphene FETs in relation to the applied tensile strain [135]. Copyright © 2011, American Chemical Society. (**d**) Schematic illustration (left side on the top: photography) and (**e**) transfer curves of CVD−grown 2D WSe_2_ FETs with [EMIM][TFSI]/PS−PMMA−PS ion gels. (**f**) VTCs of quasi−CMOS inverter consisting of 2D WSe_2_ FETs in relation to the bending radius [140]. Copyright © 2016, John Wiley and Sons. (**g**) Schematic illustration of the operating principle and (**h**) transfer curves of 2D MoS_2_ FETs employing a PSSH proton conductor. (**i**) Dynamic response of a resistor−load inverter containing 2D MoS_2_ FETs with PSSH with different frequencies [141]. Copyright © 2018, American Chemical Society.

Lee et al. [135] fabricated stretchable graphene transistors based on an ion-gel dielectric layer. The patterned graphene was used as a channel and for S/D electrodes. Poly(3,4-ethylenedixoylthiophene) oxidized with poly(4-styrenesulfonate) (PEDOT:PSS) was used as a gate electrode, which excluded the use of conventional metal electrodes. As a dielectric layer, ion gel composed of [EMIM][TFSI] and poly(styrene-methyl methacrylate-styrene) (PS-PMMA-PS) triblock copolymer was printed. As shown in Figure 8a, all the FETs with different graphene thicknesses could be operated within the low-voltage region (less than 2 V). Among the devices, the tri-layered graphene FET showed enhanced performance, with hole and electron *μ* values of 1131 and 362 cm^2^/Vs, respectively. Owing to the absence of a metal electrode and the excellent mechanical properties of the graphene and ion gel, a stretchable graphene FET could be produced (Figure 8b). There were no significant changes in the hole and electron *μ* values or the *I*_D_ and *V*_Dirac_, even with the applied strain of 5% (Figure 8c). This excellent stretchability was also verified by fabricating graphene FETs on a balloon, which maintained their electrical characteristics during stretching.

Ion gels have also been used in 2D TMD FETs. For example, Pu et al. [140] developed a CVD-grown 2D WSe_2_ FET with an ion gel consisting of [EMIM][TFSI] and PS-PMMA-PS. The fabricated 2D WSe_2_ FET showed ambipolar transport characteristics, with hole and electron *μ* values of 55 and 13 cm^2^/Vs, respectively. Combining the 2D WSe_2_ FETs with 2D MoS_2_ FETs, a CMOS inverter with maximum voltage gain values of 110 was produced. The operating voltage of the CMOS inverter was less than 2 V, suggesting the applicability of ion-gel dielectrics to complicated circuits, even if they are connected by external wires. A flexible 2D WSe_2_ FET was developed by transferring the CVD-grown WSe_2_ onto the PI substrate and drop-casting the ion-gel dielectric layer, and it exhibited clear ambipolar transport characteristics (Figure 8d,e). A flexible quasi-CMOS inverter based on 2D WSe_2_ FETs with a maximum gain of ~30 could be produced thanks to these ambipolar characteristics. As shown in Figure 8f, there was no significant degradation in the VTCs, even with the bending radius down to 0.5 mm.

In addition to ion gels, polymer electrolytes have been employed in 2D TMD FETs. Choi et al. [141] used a polyanionic proton conductor—poly(styrenesulfonic) acid (PSSH)—to gate a 2D MoS_2_ FET (Figure 8g). Before employing it with the FET devices, it was confirmed that there was no significant electrochemical reaction in the PSSH within the range of the operating voltage (<2 V). In PSSH, the protons are readily mobile, but the motion of the polyanionic backbone is limited. These imbalanced cation and anion mobilities induced the accumulation of electrons while restricting hole accumulation, leading to the prominent unipolar behavior of the 2D MoS_2_ FET. Low-voltage operation (<2 V) was achieved with a high electron *μ* of 131 cm^2^/Vs based on PSSH (Figure 8h). A lower *μ* (~45 cm^2^/Vs) was observed in the bottom-gate operation with the SiO_2_ dielectric layer despite the high operating voltage (~60 V). The temperature-dependent measurements indicated that this enhanced performance with PSSH was caused by the quenching of the phonon modes. A resistor-load inverter was demonstrated using the 2D MoS_2_ FET based on PSSH, which showed a maximum voltage gain of 4 and clear signal inversion up to 1 kHz (Figure 8i).

Although this section provides a brief introduction, the use of ion gels and polymer electrolytes is still an attractive strategy to optimize device performance and achieve low-voltage operation of 2D semiconductor FETs, as described elsewhere [149,150,151,152].

## 6. Conclusions and Outlook

This paper reviewed recent developments of emerging FET devices combining polymer gate dielectric materials and 2D semiconductors. Many of the 2D semiconductor FETs rely on inorganic dielectric materials due to their high dielectric constants and robust insulating performance [29,30]. However, polymer dielectric materials have clear advantages that make them distinguishable from inorganic counterparts, as discussed in this paper. For example, the reduced interfacial trap density of polymer dielectrics compared to oxide materials can enhance charge transport performance and the designated chemical functionalities allow for the systematic optimization of the electrical characteristics of 2D semiconductor FETs (e.g., *V*_T_). Moreover, flexible 2D semiconductor FETs have been produced by exploiting the mechanical softness of polymer materials. In addition, the unique properties of ferroelectric polymers can be applied to 2D semiconductor ferroelectric memory and NC-FET devices, and polymer films can play a role as matrices for ion-gel dielectrics to produce low-voltage 2D semiconductor FETs.

Despite these research efforts, there are still challenges and opportunities remaining for the application of polymer materials in 2D semiconductor FETs, as follows:

(1) The development of polymer materials in line with the discovery of new 2D materials is required. Polymer dielectrics can provide an optimum interface with reduced trap densities and/or chemical functionalities. In addition, relatively simple methods with mild processing conditions for polymer films allow for the fabrication of FETs with different device structures without damaging the 2D materials. Therefore, utilization of polymer dielectric materials can maximize the performance of 2D semiconductor FETs without restricting the design of materials or device structures;
(2) Large-area 2D semiconductor FET devices have rarely been produced. In this context, the thermal and chemical stability of polymer dielectric materials becomes important, as large-area growth of 2D materials typically demands high process temperatures and down-scaling is required for lithographic patterning [90,92]. Furthermore, an appropriate device structure and accompanying fabrication method need to be designed to ensure uniform electrical characteristics throughout the large area;
(3) For practical applications involving 2D semiconductor-based devices, the low-voltage operation of FETs should be secured, which requires enhancement of the dielectric constant and decreases in the thickness of the polymer dielectric layers [45]. A high dielectric constant is also important to improve FET characteristics through screening Coulomb scattering. Low-voltage 2D semiconductor FETs are rarely produced without the use of an additional inorganic dielectric layer that may restrict the mechanical properties. Ferroelectric polymers and ion gels can show high dielectric constants; however, *I*–*V* hysteresis needs to be reduced and high frequency operation should be ensured;
(4) Two-dimensional semiconductors have restricted applications due to the constraints on the process technology that can be used to control their electrical properties. Ion implantation is a common doping process technology that can regulate semiconductor electrical properties, but it causes lattice structure damage to TMDs, leading to the deterioration of their electrical properties. Consequently, there is a need to create a suitable doping process that can preserve the 2D material lattice structure while still controlling its electrical properties. To address this issue, the use of polymers as dopants to control 2D semiconductors has been reported [2,5,153], but this approach still requires accurate control of the doping degree and compatibility with atomically thin layers.

More active research and development are needed to overcome these limitations. In spite of the remaining challenges, polymer dielectric materials have various advantages and unique properties that are difficult to obtain in other materials. Therefore, we believe that polymer dielectric materials will play an important role in 2D semiconductor devices.

## Figures and Tables

**Table 1:** Summary of the materials, processing methods, device structures, and electrical characteristics for 2D semiconductor FETs based on polymer gate dielectrics.

2D Material	Dielectric Layer	Processing Method for Polymer	Gate Structure	*μ* (cm^2^/Vs)	Operating Voltage (V)	Ref.
InSe	PMMA/Al_2_O_3_	Spin-coating	Bottom gate	1055	6	[60]
MoS_2_ MoSe_2_	PS-brush/Al_2_O_3_	Spin-coating	Bottom gate	20 1.8	9 5	[61]
MoS_2_ MoTe_2_	BCB/SiO_2_	Spin-casting	Bottom gate	15.8 18.2	40	[62]
MoTe_2_	BCB/Al_2_O_3_	Spin-casting	Bottom gate	10	1	[62]
MoS_2_	CYTOP cPVP	Spin-coating	Bottom gate	43.0 42.7	20 30	[68]
3L MoS_2_ 10L MoS_2_	CYTOP/Al_2_O_3_	Spin-coating	Top gate	6.0 14.9	10	[39]
MoSe_2_	CYTOP/SiO_2_	Spin-coating	Bottom gate	16.4 (hole) 18.4 (electron)	40	[69]
MoS_2_	cPVP	Spin-coating	Bottom gate	4.7	40	[93]
MoS_2_	Al_2_O_3_/SU-8	Spin-coating	Bottom gate	141.3	40	[21]
Graphene	Parylene/SiO_2_	CVD	Bottom gate	10,600 (hole) 9800 (electron)	60	[95]
MoSe_2_	Parylene/SiO_2_	CVD	Bottom gate	118	60	[96]
WS_2_	Parylene	N/A *	Bottom gate	8.3	75	[97]
MoS_2_	P(V4D4-co-CHMA)	iCVD	Top gate	35.1	5	[106]
MoS_2_	PV3D3/Al_2_O_3_	iCVD	Bottom gate	10.4	5	[107]
Graphene	P(V3D3-co-VIDZ)	iCVD	Top gate	7200 (hole) 3800 (electron)	3	[108]

N/A *: not specified in the paper.

**Table 2:** Summary of materials and electrical characteristics of the 2D semiconductor FETs based on ferroelectric polymer gate dielectric materials.

2D Material	Dielectric Layer	*μ* (cm^2^/Vs)	SS (mV/dec.)	Operating Voltage (V)	Applications	Ref.
MoS_2_	P(VDF-TrFE)	625	N/A *	30	Ferroelectric memory	[121]
BP	P(VDF-TrFE)	131~1159	900~3300	20	Ferroelectric memory	[122]
WSe_2_	P(VDF-TrFE)	257	N/A	60	Ferroelectric memory	[123]
Graphene	P(VDF-TrFE-CFE)	3170 (hole) 1630 (electron)	N/A	10	Flexible FET	[124]
MoS_2_	P(VDF-TrFE-CFE)	51.9	N/A	10	Photodetector	[125]
MoS_2_	P(VDF-TrFE)	86.5	N/A	40	Photodetector	[126]
MoS_2_	P(VDF-TrFE)	220	300	20	Ferroelectric memory	[127]
MoS_2_	P(VDF-TrFE)	175	N/A	20	Ferroelectric memory	[129]
MoS_2_	Al_2_O_3_/metal/P(VDF-TrFE)	N/A	11.7	9	NC-FET	[132]
MoS_2_	P(VDF-TrFE)	N/A	24.2	40	NC-FET	[133]
MoS_2_	HfO_2_/P(VDF-TrFE)	−	37.2	3	NC-FET	[134]

N/A *: not specified in the paper.

## Data Availability

Not applicable.
